# Synthesis and Physical Property Characterisation of Spheroidal and Cuboidal Nuclear Waste Simulant Dispersions

**DOI:** 10.3390/ma11071235

**Published:** 2018-07-18

**Authors:** Jessica Shiels, David Harbottle, Timothy N. Hunter

**Affiliations:** School of Chemical and Process Engineering, University of Leeds, Leeds LS2 9JT, UK; d.harbottle@leeds.ac.uk

**Keywords:** inorganic synthesis, nuclear waste, caesium phosphomolybdate, zirconium molybdate, sedimentation

## Abstract

This study investigated dispersions analogous to highly active nuclear waste, formed from the reprocessing of Spent Nuclear Fuel (SNF). Non-radioactive simulants of spheroidal caesium phosphomolybdate (CPM) and cuboidal zirconium molybdate (ZM-a) were successfully synthesised; confirmed via Scanning Electron Microscopy (SEM), powder X-ray diffraction (PXRD) and Fourier transform infrared (FTIR) spectroscopy. In addition, a supplied ZM (ZM-b) with a rod-like/wheatsheaf morphology was also analysed along with titanium dioxide (TiO_2_). The simulants underwent thermal gravimetric analysis (TGA) and size analysis, where CPM was found to have a D50 value of 300 nm and a chemical formula of Cs_3_PMo_12_O_40_·13H_2_O, ZM-a a D50 value of 10 μm and a chemical formula of ZrMo_2_O_7_(OH)_2_·3H_2_O and ZM-b to have a D50 value of 14 μm and a chemical formula of ZrMo_2_O_7_(OH)_2_·4H_2_O. The synthesis of CPM was tracked via Ultraviolet-visible (UV-Vis) spectroscopy at both 25 °C and 50 °C, where the reaction was found to be first order with the rate constant highly temperature dependent. The morphology change from spheroidal CPM to cuboidal ZM-a was tracked via SEM, reporting to take 10 days. For the onward processing and immobilisation of these waste dispersions, centrifugal analysis was utilised to understand their settling behaviours, in both aqueous and 2 M nitric acid environments (mimicking current storage conditions). Spheroidal CPM was present in both conditions as agglomerated clusters, with relatively high settling rates. Conversely, the ZM were found to be stable in water, where their settling rate exponents were related to the morphology. In acid, the high effective electrolyte resulted in agglomeration and faster sedimentation.

## 1. Introduction

The Highly Active Liquor Evaporation and Storage (HALES) plant at Sellafield, UK, consolidates waste raffinates from the reprocessing of Spent Nuclear Fuel (SNF) by dissolution of the waste fission products in nitric acid, before concentrating them via evaporation [1]. The waste Highly Active Liquor (HAL) is made up of several fission products, including caesium phosphomolybdate (Cs_3_PMo_12_O_40_·xH_2_O, CPM) and zirconium molybdate ([ZrMo_2_O_7_(OH)_2_]·xH_2_O, ZM) [2], which precipitate out during temporary storage within the Highly Active Storage Tanks (HASTs) before eventual vitrification [3]. Consequently, critical research is required on non-radioactive simulants to aid behavioural understanding of the HAL precipitated dispersions. In addition, more knowledge is needed on to how the physical properties of HAL may change once processing moves to a Post Operational Clean Out (POCO) stage, where the relative concentrations of nitric acid may be diluted, potentially altering dispersion stability and other properties. 

ZM has been the focus of several studies within literature, where it has been synthesised for various applications, such as a Technetium-99m generator [4,5,6,7], a precursor for the formation of the negative thermal expansion material ZrMo_2_O_8_ [8,9] and predominately for nuclear waste based studies [10,11,12,13,14,15,16,17,18,19,20,21,22,23]. Clearfield et al. [24] were the first to publish a synthesis route for ZM to characterise its ion exchange properties. Paul et al. [21] further investigated the synthesis method, including using the addition of citric acid to alter the crystal morphology. ZM is known to cause issues within the nuclear industry, due to its mobility properties leading to potential problems with pipe blockages, for example [1]. 

There have been fewer studies investigating CPM, although there are a number of analogues that have been studied, such as ammonium phosphomolybdate, which is a potential cation-exchange material for selective recovery of Cs [25,26]. Indeed, CPM has also been synthesised itself for the same purpose, and as a photocatalyst for the photodegradation of dye pollutant [27,28,29,30]. The physical behaviour of CPM in nuclear waste HAL is of concern due to the presence of the radioactive isotopes ^134^Cs and ^137^Cs, which, if concentrated, could form potential hotspots within the HASTs [1]. Paul et al. [21] also published a synthesis route to CPM, in order to study its morphology in nuclear HAL systems, and this method was also used in the current research.

Several studies have looked into both CPM and ZM, specifically relating to the issues they cause within the HASTs, the challenge they pose to the Waste Vitrification Plant (WVP) and the in situ conversion of CPM to ZM [1,2,21,22,23,31,32,33]. Given the complexity of CPM and ZM dispersion behaviour, there is a critical need to study their synthesis and physical behaviour under a wide range of conditions. For example, there is no current information on the kinetics of CPM formation, or what impact storage temperature changes may have on growth rates and final morphology. Additionally, the main route for formation of ZM in nuclear operations is from metal substitution reactions with precipitated CPM, in current holding tanks. While it is known that these conversion reactions are very slow kinetically [21], exact time scales for ZM precipitation by this route are not known, although it has been reported that an increase in temperature and a decrease in acidity promote the conversion [31]. Different wash regents for both compounds have also been investigated with a suggestion that doping could be used to change the morphology of ZM, which has potential to be advantageous for transport or separation, depending on properties, such as sedimentation rate [1,32]. Consequently, a fuller understanding of the impact of ZM morphology on its dispersion behaviour is required.

Therefore, this study investigated a number of physical and chemical properties of synthesised non-active ZM of various morphologies and CPM, in different conditions. Additionally, the settling behaviour of these simulants was explored to understand the influence of acid concentration, which is of particular importance in the case of POCO, where the washout fluid pH and electrolyte concentration will be critical considerations. Titanium dioxide was also used, as a cheap and more easily obtainable comparison simulant, as it is a material that has been widely studied and has been previously used as a nuclear waste simulant in several studies [23,34]. 

## 2. Results and Discussion 

### 2.1. Synthesis and Formation Tracking

**Figure 1:**
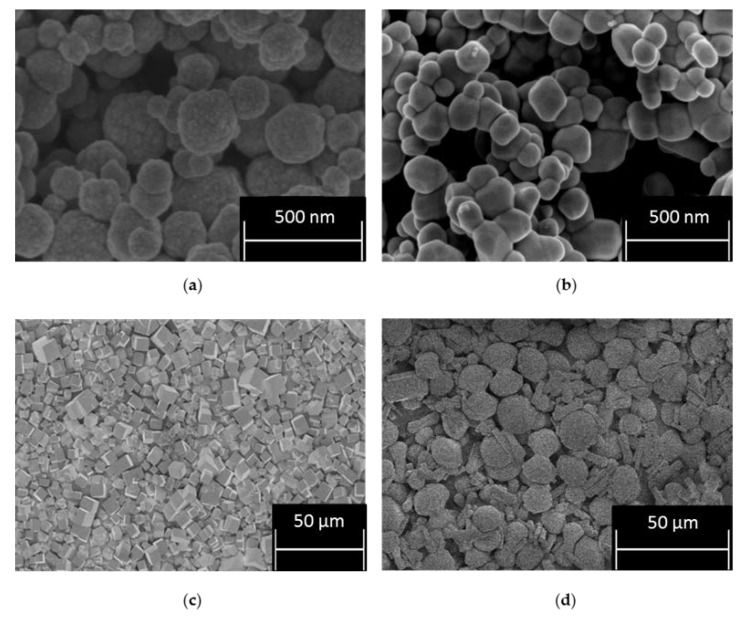
Scanning electron micrograph of: (**a**) caesium phosphomolybdate (CPM) formed at 50 °C; (**b**) titanium dioxide (TiO_2_); (**c**) zirconium molybdate (ZM-a); and (**d**) zirconium molybdate (ZM-b).

The Scanning Electron Micrograph (SEM) images in Figure 1 show the CPM, TiO_2_ and two morphologies of ZM (cuboidal and wheatsheaf) referred to as ZM-a and ZM-b, respectfully. The images for CPM and ZM-a are in agreement with those published by Paul et al. and Dunnett et al. [23,33]. CPM is known to generally exhibit a roughly spheroidal shape, consisting of agglomerated nanoclusters, whereas ZM is most commonly known to be discrete cuboidal in shape. TiO_2_ is also spheroidal in shape, consisting of bound agglomerated clusters of nanocrystallites, which appear comparable to CPM, and an appropriate comparison material, from a morphological perspective. The SEM image of ZM-b shows a mixture of both rod-like particles and a shape somewhat resembling a sheaf of wheat, hence commonly named “wheatsheaf”. The addition of citric acid in the ZM synthesis alters the cubic morphology of the particles as it binds to certain faces of the ZM that reduces their growth, and therefore the particle aspect ratio is increased [21]. What is unclear is why there is a mix of both wheatsheaf and rods formed. It is noted that the ZM-b particles used were produced at an industrial scale with different reagent conditions to the laboratory synthesised ZM-a, and it is likely that the final morphology of the particles is sensitive to various factors, such as concentration of the citric acid reagent added, potential trace contaminants or even different precursor materials. As the chemistry of the HAL and the conditions of the HASTs are largely unknown, it is not unreasonable to suggest a mix of ZM morphologies could be present, and therefore ZM-b may represent a more realistic simulant than the well-defined cuboidal ZM-a. Additionally, depending on the morphology of the ZM and the properties it exhibits, doping the HAL to promote a morphology change before POCO could be advantageous [1]. This complexity re-enforces the need to characterise various morphologies of ZM to investigate the potential differences in physical and chemical properties.

**Figure 2:**
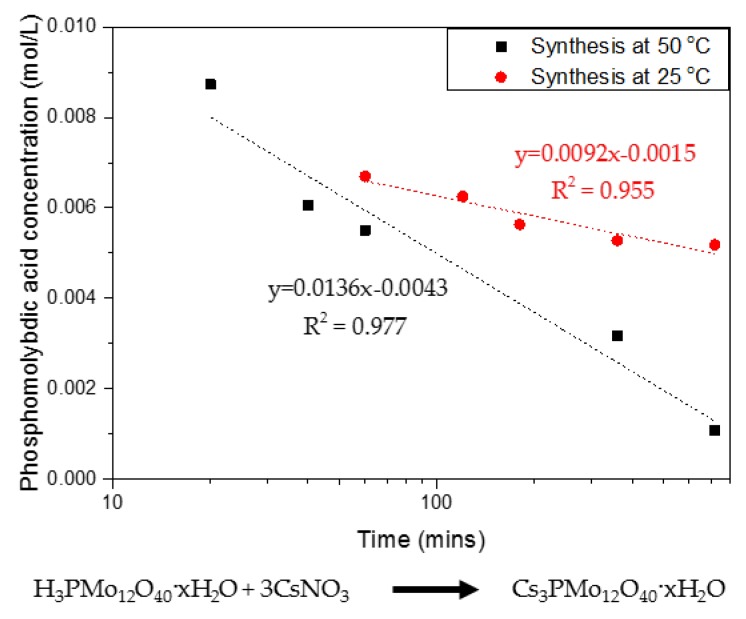
Rate of reaction showing first-order kinetics for the co-precipitation reaction of caesium nitrate and phosphomolybdic acid forming caesium phosphomolybdate (CPM) at 25 °C and 50 °C with corresponding reaction equation.

The synthesis of CPM involves a double replacement reaction with two reactants, namely phosphomolybdic acid (H_3_PMo_12_O_40_·xH_2_O) and caesium nitrate (CsNO_3_), as discussed in detail by Paul et al. [21]. Phosphomolybdic acid is Ultraviolet–Visible (UV–Vis) active, which allowed the precipitation kinetics of CPM to be tracked via measuring the phosphomolybdic acid decrease in concentration over time. Figure 2 shows the rate of reaction curves determined for the CPM synthesis conducted at both 25 °C and 50 °C, through tracking of the phosphomolybdic acid concentration. It was found to be a first-order reaction in respect to phosphomolybdic acid, which is in excess to the caesium nitrate, giving a rate constant of 0.04 min^−1^ for the reaction at 25 °C and of 0.09 min^−1^ for the reaction at 50 °C. This result demonstrates the fast kinetics at which CPM is formed within laboratory conditions, and, while published synthesis routes often quote total reaction times of ~48 h [21], it is clear that the actual reaction is almost at completion within 1 h at the higher temperature. While the reaction environment will differ within the HASTs, average temperatures are kept within 50–60 °C, suggesting a similar reaction rate to that found at 50 °C could be feasible. It is also noted that, due to the high relative proportions of caesium within the fission products, it is expected that CPM will form easily and in high amounts. 

**Figure 3:**
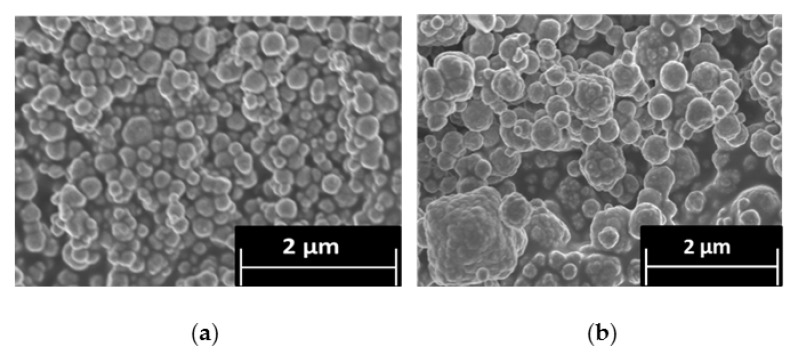
Scanning electron micrograph of: (**a**) caesium phosphomolybdate (CPM) synthesised at 25 °C; and (**b**) CPM synthesized at 100 °C.

CPM was also synthesized at 100 °C to investigate the effect of temperature differences on its morphology. Figure 3 shows SEM images of the synthesized CPM at both 25 °C and 100 °C. The CPM synthesized at 25 °C compared well to the CPM synthesized at 50 °C (Figure 1a), suggesting that application of heat between 25 °C and 50 °C does not alter the final particle morphology significantly (although the kinetics of the reaction are slower, as shown in Figure 2.). For the CPM synthesized at 100 °C, it was found that, although spheroidal particles were also formed, there was an increase in large agglomerates and a greater range of particles varying in size and in shape, compared to the CPM synthesized at 25 °C and 50 °C. A potential reason for the differences may be that the faster reaction kinetics that occur with increase in temperature means that there is less time for the nanocrystallite clusters to form into self-similar spheroidal particles through diffusion interaction. As seen with the difference in kinetics between 25 °C and 50 °C, if there is a similar increase in the rate of precipitation at 100 °C, the CPM will precipitate almost instantaneously, leading to larger and more disordered clusters. Additionally, at 100 °C, it would be expected that formed CPM would be less stable, as it is known to be a temperature range in which its breakdown should begin to occur [30]. Hence, precipitates may partially re-solubilise, especially outer surfaces, leading to fusion of nanocrystallite clusters.

**Figure 4:**
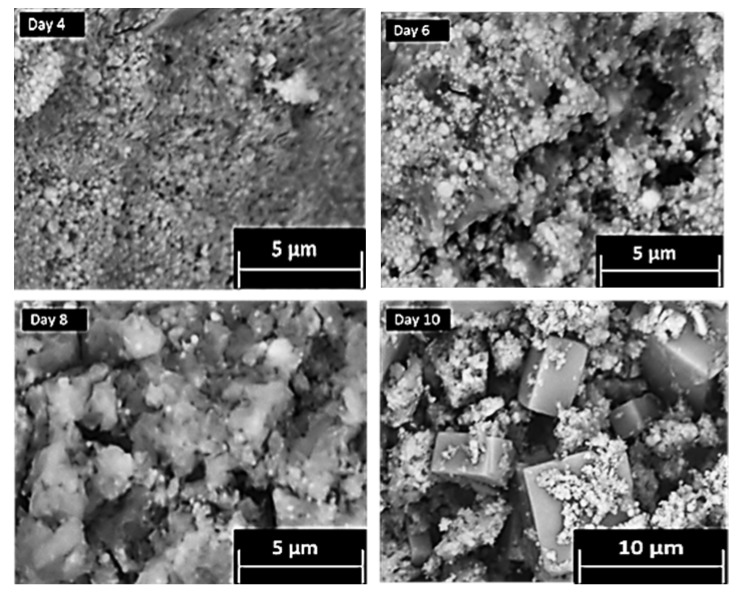
Scanning electron micrographs taken at various times during the tracking of zirconium molybdate (ZM-a) synthesis from a caesium phosphomolybdate (CPM) precursor over 10 days.

The conversion of CPM to ZM-a was also qualitatively tracked, via SEMs of intermediate structures over a period of ten days, as presented in Figure 4. Through Days 0–6, it was observed that the particles remain predominantly spheroidal in their morphology and nanometre in size, representative of CPM. In addition, the solids all remained yellow in colour, visually a characteristic expected for CPM [21]. For the solids precipitating out from Day 8 to Day 10, there appeared to be a mix of yellow and white solids suggesting that ZM-a was beginning to form (Supplementary Materials, Figure S1 shows the colours of both pure CPM and ZM-a solids). The SEMs taken at Days 8 and 10 also suggest this, as the cuboidal micrometre particles that would be expected of ZM can be seen to appear, in addition to the spheroidal CPM particles coating the ZM-a. Once Day 10 was reached, the solids were washed with 1 M ammonium carbamate which dissolved the CPM particles [1], resulting in the clear cuboidal particles, as seen in Figure 1c. This sequence of SEM images demonstrates the length of time it takes for ZM to transform from CPM in a highly controlled environment, and it is evident that the conversion yield from CPM to ZM remains low (estimated to be ~30–40%). In comparison, as Figure 2 demonstrates, the CPM forms rapidly (within hours), suggesting that there may be a higher concentration of CPM in contrast to ZM within the HASTs. However, tank conditions and specific compositions of the HAL within the tanks are variable, and thus the ratio of CPM:ZM is extremely difficult to predict. This issue highlights the importance of understanding both systems individually, especially considering the amount of time that HAL is left in the tanks, which is often in the order of months.

### 2.2. Chemical Composition 

**Figure 5:**
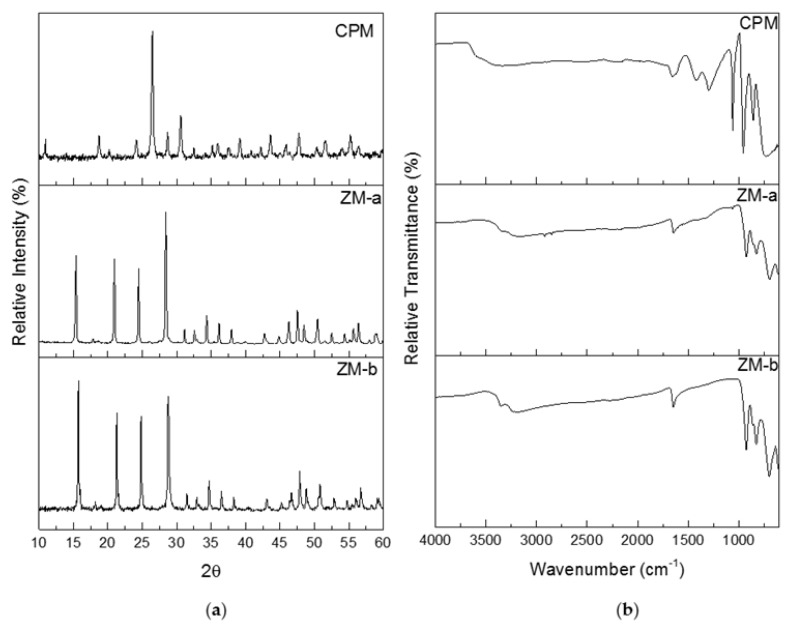
(**a**) Powder X-ray Diffraction patterns for caesium phosphomolybdate synthesised at 50 °C (CPM), and zirconium molybdate (ZM-a and ZM-b). (**b**) Infrared spectra of CPM synthesised at 50 °C, ZM-a and ZM-b.

Figure 5a shows the powder X-ray diffraction (PXRD) patterns for CPM, ZM-a and ZM-b. Both the ZM patterns were compared to The International Centre for Diffraction Data (ICDD) online database, where they correlated with the ICDD number 04-011-0171. ZM morphologies are reported to crystallise as a body-centred tetragonal lattice with space group *I41cd*, lattice parameters a = b = 11.45 Å, c = 12.49 Å and angles *α* = *β* = *γ* = 90^o^. Although the morphologies of ZM-a and ZM-b are quite different, the PXRD patterns show their bulk crystal structure remains the same. The PXRD patterns for ZM-a and ZM-b also agree with those published within the literature [21,24]. Whilst the pattern for CPM was not found in the ICDD database, it is in good correlation to the patterns for CPM found within the literature [21,27]. CPM is reported to crystallise in a cuboidal lattice with space group *Pn*-3*m*, lattice parameters a = b = c = 11.79 Å and angles *α* = *β* = *γ* = 90^o^.

Infrared analysis was used as a fast method for fingerprinting the synthesised compounds, following methods previously detailed in the literature [11,24]. Figure 5b, presents the Fourier Transform Infrared (FTIR) spectra for the synthesised CPM, ZM-a and ZM-b. The spectrum published by Rao et al. [11] is in good agreement to the ZM spectra shown in Figure 5b. The spectra for the ZM samples shows a band between 3000 cm^−1^ and 3300 cm^−1^, which is representative of the O–H group, with the band at 1600 cm^−1^ representing the O–H–O bonds. The “fingerprint” region is below 1000 cm^−1^ corresponding to metal to oxygen groups. If the sample was to be anhydrous, the IR spectrum would differ with no bands within 400–700 cm^−1^ [11]. The ZM-b spectrum differs slightly from the ZM-a spectrum with more intense bands around 1000 cm^−1^, potentially equated to the presence of more bound water molecules present in the structure. For the CPM spectrum, the higher region, from 1250 cm^−1^ upwards, is largely similar to the ZM spectra (representing the O–H and O–H–O groups with slight intensity differences). The CPM spectrum published by Ghalebi et al. [30] is in good agreement to the CPM spectrum published here. The “fingerprint” region (<1000 cm^−1^) highlights clear differences in intensity from the ZM, due to the main metal bonding, indicating IR probe analysis may potentially be useful as an in situ technique to determine compositional differences, in CPM/ZM mixtures.

**Figure 6:**
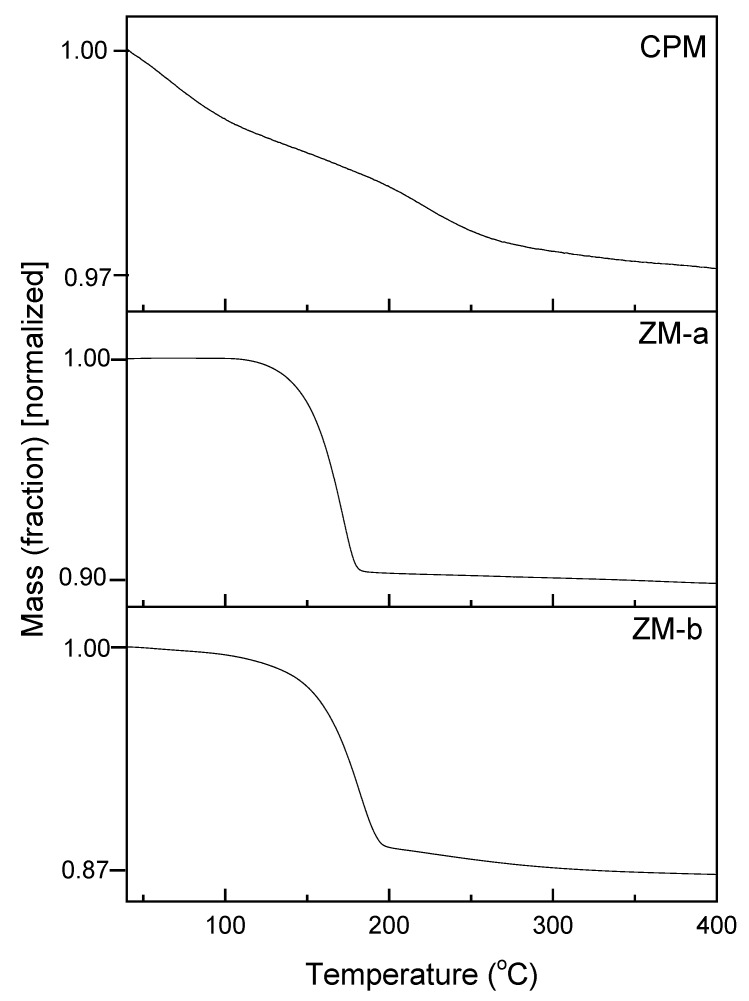
Thermogravimetric analysis curves of caesium phosphomolybdate synthesised at 50 °C (CPM) and zirconium molybdate (ZM-a and ZM-b). CPM shows a loss of 13 moles of water, ZM-a a loss of 3 moles of water and ZM-b a loss of 4 moles water.

Figure 6 presents the thermal gravimetric analysis (TGA) plots of CPM, ZM-a and ZM-b, over the temperature range from 30 °C to 400 °C. For CPM, the water loss begins below 100 °C and continues until ~400 °C, with a total mass loss equating to 13 moles of water, therefore resulting in a chemical formula: Cs_3_PMo_12_O_40_·13H_2_O. This value is in good agreement with the reported literature values, which are generally between 9 and 14 moles of water, depending on the drying method chosen [24]. In the present study, CPM was dried using an oven at 70 °C for 12 h. Therefore, it is possible some of the initial water loss could potentially have been strongly adsorbed bound water, or water with extremely low binding energy, although, given the length of drying time, it is not assumed to be from any free water. In comparison, the dehydration process for both ZM morphologies had a clearer start and end temperature. For ZM-a, the mass loss starts around 100 °C and stops just before 200 °C, equating to three moles of water and a chemical formula of ZrMo_2_O_7_(OH)_2_·3H_2_O, which is the same value reported in literature [17]. The dehydration process of ZM-b is similar, but appears to begin with a slightly slower rate and lower temperature, while again the mass loss stops just before 200 °C. This value equates to a loss of four moles of water, showing a difference in bound water content between the ZM-a and ZM-b samples. This may be a result of the citric acid incorporation, although, again, differences in reaction conditions may also be a cause. The extra water present in ZM-b is also consistent with the slight variation in the FTIR spectrums (Figure 5b) with the more intense band <1000 cm^−1^ for ZM-b in comparison to ZM-a.

### 2.3. Size, Stability and Settling Behaviour 

**Figure 7:**
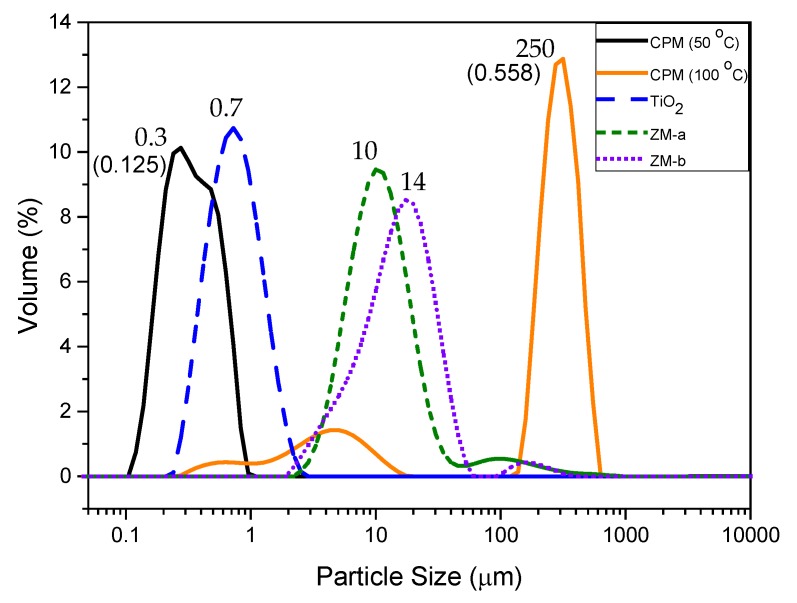
Particle size distributions of caesium phosphomolybdate (CPM) synthesised at 50 °C and 100 °C, titanium dioxide (TiO_2_) and zirconium molybdate (ZM-a and ZM-b). Corresponding D50 values shown by each relevant peak. Polydispersity index values for CPM at 50 °C and 100 °C shown in brackets.

The particle sizes of all simulants are shown in Figure 7, as a relative volume percentage versus size distribution. The D50 value of CPM synthesised at 50 °C is 300 nm, which corresponds well with the SEM images of CPM nanoclustersnshown in Figure 1a. In comparison, the CPM synthesised at 100 °C has a significantly higher D50 value of 249 μm, which is even larger than evident from the SEM images (Figure 3b). Although individual crystallite sizes appear (via SEM) to be fairly similar to those formed at lower reaction temperatures, it is clear they cluster to a much greater degree and become extremely agglomerated, resulting in the high D50 value. The secondary smaller peak representing the finer particles ranging between 300 nm to 20 μm, likely represents both individual particles and smaller agglomerates. The poly dispersity index (PDI) for CPM synthesised at 100 °C is 4.5 times larger than the CPM synthesised at 50 °C, which is attributed to the bimodal distribution of the CPM synthesised at 100 °C. 

The TiO_2_ was expected to be slightly larger than the CPM particles synthesised at 50 °C, due to agglomeration, as evidenced in the SEM images (Figure 1b) and confirmed by the D50 value of 700 nm for TiO_2_ in comparison to the CPM 50 °C D50 value of 300 nm. For the ZM-a simulant, the cuboidal particles are shown to be around thirty times larger than the CPM with a D50 value of 10 μm. Unsurprisingly, when comparing SEM images (Figure 1d), ZM-b displays a larger D50 value than ZM-a with a value of 14 μm, although caution must be taken with light scattering estimations of any non-spherical particles [22]. In comparison to the literature, the CPM synthesised at 50 °C particle size is within the expected range, [21] while the D50 for the ZM-a is slightly larger than previously reported [33].

**Figure 8:**
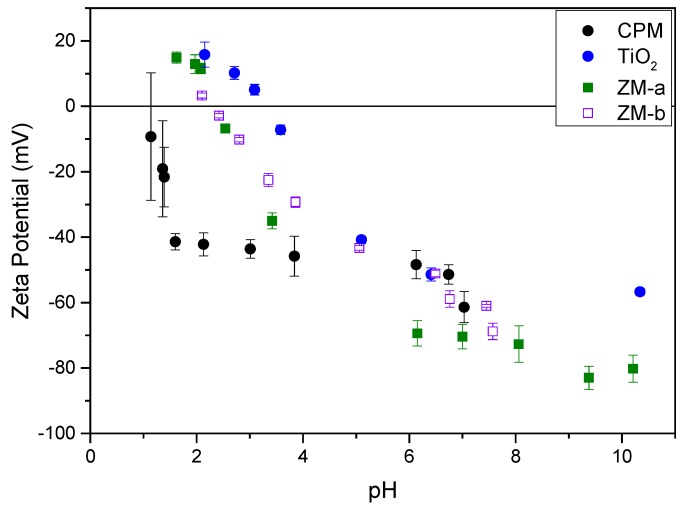
Zeta potential curves for caesium phosphomolybdate (CPM), titanium dioxide (TiO_2_) and zirconium molybdate (ZM-a and ZM-b) measured at concentrations of 1000 ppm in 10^−4^ M potassium nitrate (KNO_3_) solution.

The zeta potentials of all simulants as a function of pH are shown in Figure 8. The Isoelectric Point (IEP), was around pH 2.5 for both ZM-a and ZM-b, which is similar to that found by Paul et al. for cuboidal and rod-like ZM particles [22]. For CPM, the IEP could not be obtained confidently due to the observed error at very low pH, likely because of the high effective counterion concentration. However, through extrapolation, the IEP appears to be in the region of pH 1–1.5, and again similar to values previously reported by Paul et al. [22]. TiO_2_ had the highest IEP at ~pH 4, which compares well to previous literature on measurements of anatase and rutile mixtures [35,36]. The zeta potential data largely suggest that, in low pH conditions, such as those experienced in the HASTs, the ZM species will be positively charged, while the CPM may be close to an uncharged state. However, the high acid concentration in the processing environments will mean that there is a high effective electrolyte concentration (resulting from acid counterions), collapsing the electric double layer around the particles, despite any native charge at low pH. Thus, it is important to study dispersion stability in both acid and water environments (that latter of which may represent conditions in POCO).

Further, previous reported work by Paul et al. [22] indicated that the equilibrium pH for CPM and ZM dispersions in water may be significantly reduced over time, due to potential hydride reactions from the bound water. Therefore, the equilibrium pH after 48 h of 4 vol% dispersions was measured for all HAL simulants. The pH for CPM and ZM-b was ~1.5, while for ZM-b was slightly higher at ~2.4. Therefore, even in pure water environments, the zeta potential of the simulants may be altered, affecting their stability. It would appear from the equilibrium pH measurements that ZM-b and ZM-a will be likely positively charged (although the ZM-a may be close to its IEP) while CPM will be weakly negatively charged and approaching its IEP. It is noted that the pH of titania dispersions in water was close to neutral, although very slightly acidic due to the use of deionised Milli-Q water (~pH 5.5–6).

**Figure 9:**
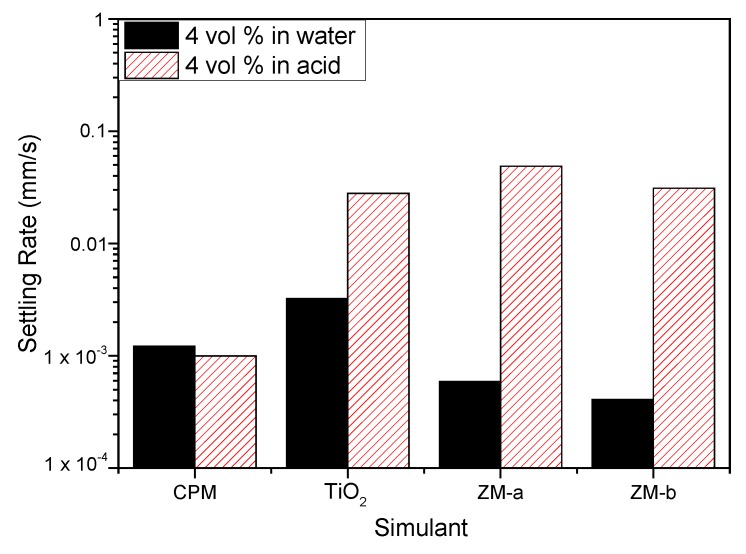
Settling rates for caesium phosphomolybdate (CPM), titanium dioxide (TiO_2_) and zirconium molybdate (ZM-a and ZM-b) at 4 vol% concentration in both water and 2 M HNO_3_ environments.

Figure 9 shows the settling rates for all the simulants in both water and 2 M HNO_3_ at 4 vol% (assumed to be close to relevant concentration conditions). Both ZM-a and ZM-b sediment at considerably higher rates in the 2 M HNO_3_, indicating that the high effective electrolyte conditions lead to significant coagulation of the dispersions, likely from the collapse of the electric double layer. For the TiO_2_ dispersions, sedimentation rates are also enhanced in acid (although to a lower degree) and, importantly, the rate in water is greater than either of the larger ZM species, suggestive also of a degree of coagulation in water conditions. While the zeta potential data indicated good stability at neutral pH, the overall values measured are an average for the mixed anatase/rutile particles, and it is known that for the anatase phase, the expected IEP is around neutral [22] (while pure rutile is ~3–4). Therefore, some degree of heterogeneity in surface charge would be expected, leading to some partial dispersion instability, which is evident from the settling data.

For CPM, the difference in settling rates is minimal for the two conditions, which would suggest similar levels of dispersion stability. As it was assumed from the equilibrium pH of CPM in water that dispersions may be close to the IEP in these conditions, the similarity of settling data also in acid indicates potential coagulation is occurring in both conditions. While the sedimentation rates for CPM are lower than for other species in acid, it is noted they have the smallest particle size. In addition, any comparison between species must be made with caution, as the 4 vol% dispersions will be within the hindered settling regime, and cannot be associated directly with expected Stokes settling velocities.

**Figure 10:**
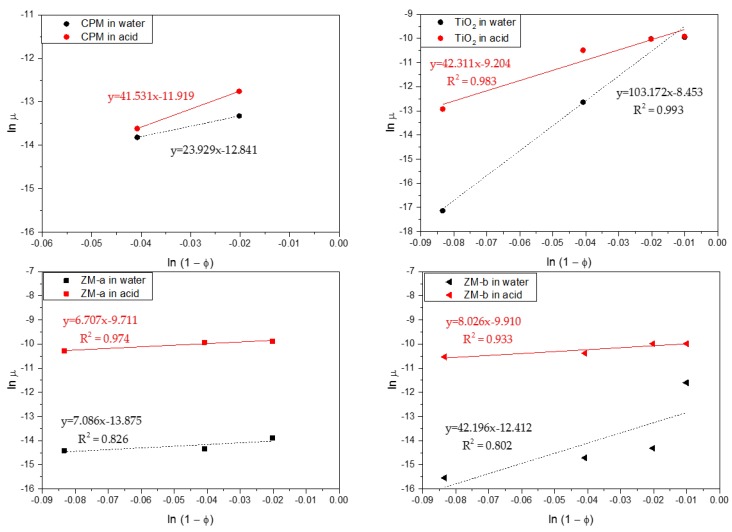
log–log linearised settling rates versus porosity (1 − Φ) for caesium phosphomolybdate (CPM), Titanium dioxide (TiO_2_) and Zirconium molybdate (ZM-a and ZM-b) in both water and 2 M HNO_3_ environments. Dashed lines indicate power-law fits.

For more quantitative analysis, sedimentation of the simulants for a range of concentrations in both water and acid were analysed using the Richardson-Zaki (RZ) power-law hindered settling model, with data presented in Figure 10 [37]. Here, the natural log of the linear settling rates *ln*(*u*) are given versus the log of the porosity *ln*(1) − Φ). Exponent values associated with the fits can aid understanding on the coagulation of the simulants, in addition to the influence of particle shape. For non-agglomerated spherical dispersions, an exponent of ~4.65 would be expected [37,38]. For spheroidal CPM, its exponent values were 23.93 and 18.05, in water and acid, respectively, while for the TiO_2_, its exponents were 103.17 and 42.31. These values are an order of magnitude, or more, greater than for spherical systems, inferring a high degree of aggregation, which is consistent with the 4 vol% settling data discussed in Figure 9. It is interesting that the TiO_2_ exponent value in water is considerably higher than in acid, even though settling rates are lower. This behaviour may indicate that while agglomerates are smaller in water, they have a more open structure, which increases hindered settling effects. 

For the ZM-a and ZM-b simulants, both show similar trends across the concentration regime measured, with their settling rates in acid being much faster than that in water, consistent with the single 4 vol% data in Figure 8. Therefore, for potential POCO environments, lower acidity wash waters may aid in stabilising ZM dispersions, reducing issues of sedimentation on transfer, whereas effects on CPM will likely be minimal. There are however some interesting differences in the power-law exponent values, especially in water. The exponent value for ZM-a in water is 7.09 while for ZM-b it is 19.48. These values are higher than expected for spherical particles, although the slow settling rates and zeta potential data suggest high stability. It is assumed that, similar to studies on stable non-spherical particles [39,40,41,42], the high exponent values occur from the enhanced drag due to their shape. Indeed, shape factor may help explain the higher value for ZM-b, as the orientation of the elongated wheatsheaf/rod-like particles may have an additional effect on the drag, which will likely be greater if they adapt a flat confirmation [43,44]. The exponent values are both similar to each other in acid, and notably less than the ZM-b in water. It may be the aggregated ZM clusters actually have a reduced drag in comparison to the elongated and stable ZM-b particles.

The zero concentration intercept from the fits in Figure 10 were used to estimate the free settling velocities of the simulants in each system. Stoke’s law was then utilised to determine what spherical equivalent size, the particles would be expected to be [22]. For CPM and TiO_2_ in both water and acid, the calculated sizes were much larger than their measured D50 mediums from Figure 7 (at ≥1 µm), which is consistent with the hypothesis that both systems are coagulated in all conditions. In comparison, for both ZM-a and ZM-b systems, the calculated particle sizes were much smaller than their measured D50 sizes (at between 1 µm and 8 µm). Estimated size values from the ZM-a and ZM-b acid settling data were however much larger (>15–30 µm) again consistent with coagulation.

The low estimates sizes for ZM in water may be due to the fact that simple Stoke’s law calculations makes several assumptions that do not apply to the ZM samples directly, such as particle sphericity and monodispersity. Considering the morphology of both ZM simulants, their representative drag coefficient will be significantly larger than that of a sphere, and Stoke’s law will likely lead to underestimations of size, as is evident from the values derived from Figure 10. Additionally, the presented settling data were taken at a single threshold of 40%. A single threshold represents a certain fraction of the particles, but it does not capture complete settling data for polydisperse systems, such as the ZM. For example, when ZM-b settling data were analysed at a range of thresholds, calculated linear settling rates vary by almost an order of magnitude (comparing 10% and 80% thresholds). While the 40% threshold was chosen as a fixed value to allow comparison of all samples, it appears this likely correlates to a fraction of the dispersion that is under the medium sizes. Therefore, caution should be taken when extracting sizes from centrifugal settling data. 

## 3. Materials and Methods 

### 3.1. Synthesis and Materials

The synthesis routes for both CPM and ZM-a were based upon the methods published by Paul et al. [21]. Phosphomolybdic acid and caesium nitrate were mixed at a 1 to 3 molar ratio with equal volumes in 2 M HNO_3_ at 25 °C, 50 °C and 100 °C over a 12 h period with constant stirring to form the yellow precipitate CPM. ZM-a was then formed from the CPM synthesised at 50 °C with the addition of zirconyl nitrate in 6 M HNO_3_ at a 1 to 1 volume ratio, at a temperature of 100 °C and constant stirring for 10 days before washing with 1 M ammonium carbamate. The reactants used for these synthesis methods are given in Table 1. In addition, the National Nuclear Laboratory (NNL) provided a simulant of ZM (ZM-b) industrially synthesised by Johnson Matthey (Royston, UK). This sample had a mix of rod-like and wheatsheaf morphology, resulting from the addition of citric acid during the synthesis process. While the overall synthesis procedure is similar to that detailed by Paul et al. [21], where rod-like particles were formed, the exact reagent conditions and concentrations for the industrial sample are unknown. Titanium dioxide (TiO_2_) in its anatase/rutile mixed form was purchased from Venator Materials PLC (formally Huntsmans Pigments Ltd., Wynyard, UK) with product code TS46424 to be used for comparative purposes and as a standard for some preliminary experiments. 

### 3.2. Ultraviolet-Visible Spectroscopy 

To track the synthesis of CPM, a UV-Vis spectrophotometer Lambda XLS (PerkinElmer, Waltham, UK) was utilised to track the concentration of one of the reactants; phosphomolybdic acid, as the other reactant (caesium nitrate) was found to not be significantly UV-Vis active. Before the reaction was conducted, a calibration curve was generated for various concentrations of phosphomolybdic acid versus their absorbance taken at wavelength 458 nm. The calibration can be seen in the Supplementary Materials, Figure S2. Once the reaction had begun, regular aliquots of the solution were taken at varying time periods, which were then diluted within 2 M HNO_3_ and centrifuged to remove any CPM solid formed before the remaining supernatant underwent UV-Vis spectroscopy. The absorbance value of the supernatant was then compared to the calibration curve previously taken for phosphomolybdic acid, to determine its concentration at that particular time. The corresponding concentrations were plotted against the time they were taken and the gradient taken in order to determine the rate constant. 

### 3.3. Particle Shape, Density and Size Characterisation 

Particle shape analysis was completed through the use of a SU8230 scanning electron microscope (SEM, Hitachi, Krefeld, Germany). The solid samples were prepared using a carbon based adhesive disk, in which dry ground up powder was placed before being platinum coated. This SEM was also used to take the images for the ZM-a morphology tracking; daily aliquots were taken from the reactor, and then centrifuged before the supernatant was removed and the solid dried before imaging. 

A Mastersizer 2000 (Malvern, Worcester, UK) was used to determine the particle size distribution for the simulants. For each sample, a small amount of solid was allowed to form a dispersion within distilled water before being added in the Mastersizer until the required transmission value was met. It is crucial that the sample be fully dispersed in order to achieve the most accurate representative sizing measurement, avoiding agglomerates of material which would give inaccurate results. Each sample was then measured 10 times over 10 s and an average was taken. The D50 value was determined by the Mastersizer as the 50th percentile, therefore is was inclusive of any bimodal distribution.

An AccuPycTM 1330 Pycnometer (micromeritics, Norcross, GA USA) was used to take density measurements via gas pycnometry. The solid simulants with a known mass were placed into the instrument then using the pressure change of helium in a known calibrated volume the density of the simulants was determined. The following densities were determined: CPM 3.82 g/cm^3^, TiO_2_ 4.23 g/cm^3^, ZM-a 3.41 g/cm^3^ and ZM-b 3.41 g/cm^3^.

### 3.4. Power X-ray Diffraction, Infrared Spectroscopy and Thermogravimetric Analysis

A D8 X-ray Diffractometer (Bruker, Coventry, UK) was used to measure the crystalline structure of the samples with an electron beam of 40 kV. The copper source (Cu K*α*) has a wavelength of 1.54 Å, energy of the radiation source was 1.6 kW, and measurements were taken over the 2θ range 10–60° with a step size of 0.032° 2θ and a scan speed of 0.2 s per step. The raw data for the diffraction pattern were then extracted and normalised for comparison to The International Centre for Diffraction Data (ICDD) online database. 

The functional groups of the simulants were determined through Fourier Transform Infrared (FTIR) spectroscopy, carried out using a Nicolet iS10 FTIR spectrometer (ThermoFisher, Waltham, UK) with a ZnSe ATR attachment, at a resolution of 4 cm^−1^ and 64 scans. Dry power samples were placed upon the sample holder before being clamped into place and the spectroscopy conducted. The spectra were then analysed to identify the functional groups.

Thermal dehydration of the simulants (used to determine the amount of bound water) was determined via thermogravimetric analysis (TGA), using a TGA/DSC 1100 LF (Mettler Toledo, Leicester, UK). For each test, a 0.1 g sample was inserted, and heated using a temperature profile from 30 °C to 400 °C at a heating rate of 10 °C·min^−1^ under a nitrogen atmosphere. The mass loss over this time period was then converted to the amount of water molecules lost for each simulant.

### 3.5. Zeta Potential Measurements and Sedimentation Experiments

The zeta potential of the simulants at various pH values were determined using a Zetasizer Nano ZS (Malvern, Worcester, UK) which directly measures the electrophoretic mobility and then uses this to calculate the zeta potential via an internal algorithm. Dispersions were prepared with concentrations of 1000 ppm in a 10^−4^ M potassium nitrate (KNO_3_) solution. Nitric acid (HNO_3_) and potassium hydroxide (KOH) solutions between 0.01 and 0.1 M were used to adjust the pH of the samples. For every sample, five measurements were taken and repeated on fresh samples 2 or 3 times, where an average of these results is presented. The Malvern Zetasizer Nano ZS was also used to calculate the polydispersity index—a parameter determined from a Cumulants analysis of an intensity–intensity autocorrelation function.

A LUMiSizer^®^ (LUM GmbH, Berlin, Germany)was used to study the dispersion settling stability, where sedimentation studies for the simulants were conducted in triplicate (with an average standard deviation of 5% of the mean value) with an average of the results taken, in both deionised water and 2 M HNO_3_. The centrifuge speed was set between 500 and 2000 RPM (depending on the sample) at 25 °C and transmission profiles taken every 10 s with the total number of profiles equalling 255. The LUMiSizer measures the solids settling rate by centrifugation using LED light sources that emit light at different wavelengths to produce-transmission profiles at set time intervals. From the produced transmission profiles, a threshold of 40% was chosen and the data converted to give suspension height vs. time, where the linear zone settling rate for each simulant at that RPM was determined (Figure S3 in Supplementary Materials shows a raw transmission profile with corresponding suspension height vs. time). A threshold of 40% was chosen as on analysis it was deemed the best representative threshold allowing for comparison of all samples. It should be noted as a limitation it represents a certain fraction of the particles at a certain size, and a single threshold cannot capture the full behaviour of a polydisperse suspension. The sedimentation rates were then back calculated to estimate the settling rate at normal gravity, assuming linear dependence on RCA, using Equations (1) and (2). Here, *RCA* is relative centrifugal acceleration, *r* is the radius of the instrument plate where the dispersion is housed (in cm) and *RPM* is the revolutions per minute of the experiment [45].

(1) $$RCA=1.1118\times 10^{-5}\times r\times RPM^{2}$$

(2) $$\frac{Measured\;velocity}{RCA}=gravity\;velocity$$

## 4. Conclusions

Research was conducted on non-active simulants of two known precipitated fission waste products found in nuclear fuel reprocessing: caesium phosphomolybdate (CPM) and zirconium molybdate (ZM). Both CPM, with spheroidal particle shape (formed from agglomerated nanoclusters), and ZM-a, with a cuboidal morphology, were successfully synthesised and characterised using SEM, PXRD, IR and TGA. In addition, ZM-b with a rod-like/wheatsheaf morphology was also characterised, along with titanium dioxide (a commercially available alternative, morphologically similar to CPM). The reaction kinetics of CPM precipitation was investigated at various temperatures, finding it to be a first-order reaction with respect to phosphomolybdic acid, and able to form at a range of temperatures, although size and stability properties begin to change at ~100 °C. While the kinetics of CPM synthesis was very fast, ZM formation from CPM precursor substitution was slow, being observed to convert partially only after ~10 days. The ease of formation of CPM compared to ZM suggest that within the HASTs there could be a larger proportion of CPM in comparison to ZM. Additionally, as the formation of ZM is sensitive to the effects of additives, it is likely that any ZM formed will contain a range of morphologies. 

The dispersion stability of the simulants in water and 2 M nitric acid was observed by comparing zeta potential and pH measurements with centrifugal sedimentation analysis. All simulants were found to have low IEP values; however, acid group leaching reduced the natural pH of water suspensions to around or below these values. Therefore, in low pH conditions such as those experienced within the HASTs, the waste products are likely to be unstable and coagulate. The CPM concentration dependence on the settling rate was found to be more pronounced due to coagulation in both water and acid environments, which were qualitatively similar to the titania. The ZM-a and ZM-b conversely appeared stable with low settling rates in water that significantly increased in acid (assumed to be caused by coagulation from the collapse of the electric double layer). Overall, results highlight the complex morphology and chemistry of these precipitated nuclear wastes, and imply their stability may be critically altered, depending on changes in acid levels as waste treatment moves to a post operational clean out phase. 

## Figures and Tables

**Table 1:** List of reagents used for the synthesis of both caesium phosphomolybdate (CPM) and zirconium molybdate (ZM).

Material	Formula	Purity	Supplier
Phosphomolybdic acid hydrate Caesium nitrate Nitric acid Zirconyl nitrate	H_3_PMo_12_O_40_ CsNO_3_ HNO_3_ ZrO(NO_3_)_2_	Solid—80% Solid—99.9% Solution—70% Solution—35 wt. % in dilute HNO_3_	Acros Organics Aldrich Fisher Scientific Sigma-Aldrich

## Supplementary Materials

The following are available online at http://www.mdpi.com/1996-1944/11/7/1235/s1. Figure S1. Images of: (**a**) caesium phosphomolybdate (CPM) formed at 50 °C displaying a yellow coloured solid; and (**b**) zirconium molybdate (ZM-a) displaying a white coloured solid. Figure S2. UV-Vis calibration curve for phosphomolybdic acid at various concentrations. Figure S3. Raw LUMiSizer settling data showing transmission profile taken at 40% converted to suspension height vs. time, for zirconium molybdate (ZM-a) at 4 vol% in water at 500 rpm.
