# Supplementary material for: Synthesis and Physical Property Characterisation of Spheroidal and Cuboidal Nuclear Waste Simulant Dispersions
Source: Materials (Basel). 2018 Jul 18;11(7):1235. doi: 10.3390/ma11071235 (PMC6073122; doi:10.3390/ma11071235)
Supplement: Supplementary file 1 [file materials-11-01235-s001.pdf]

Supplementary Materials:

Synthesis and Physical Property Characterisation of Spheroidal and Cuboidal Nuclear Waste Simulant Dispersions

Jessica Shiels *, David Harbottle and Timothy N. Hunter *

School of Chemical and Process Engineering, University of Leeds, Leeds, LS2 9JT, UK; d.harbottle@leeds.ac.uk (D.H)
*Correspondence: pmjas@leeds.ac.uk (J.S.); t.n.hunter@leeds.ac.uk (T.N.H.)

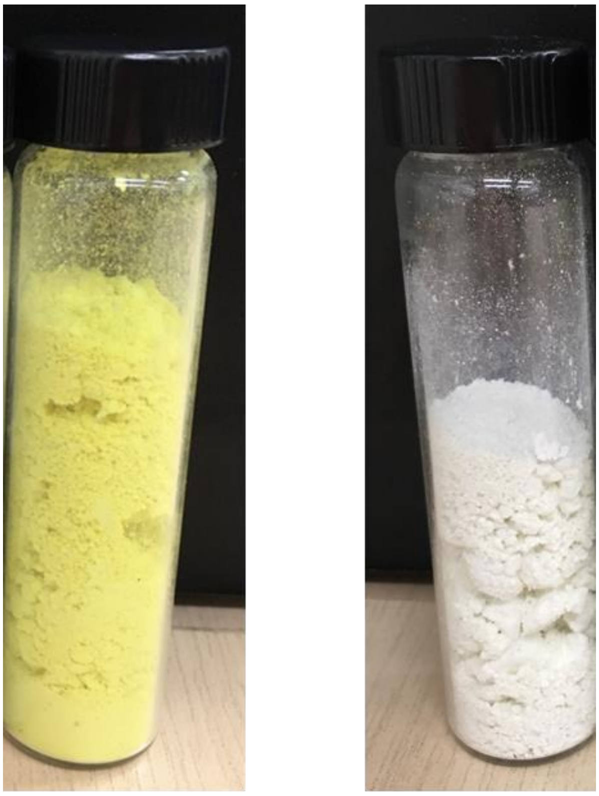

(**a**) (**b**)

Figure S1. Images of (**a**) caesium phosphomolybdate (CPM) formed at 50 °C displaying a yellow coloured solid and (**b**) zirconium molybdate (ZM-a) displaying a white coloured solid.

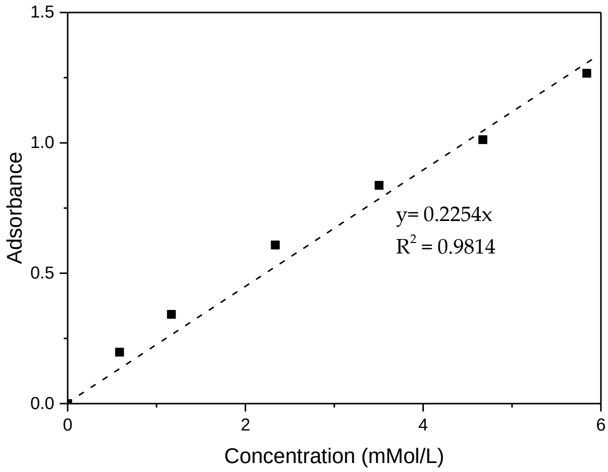

Figure S2. UV-Vis calibration curve for phosphomolybdic acid at various concentrations.

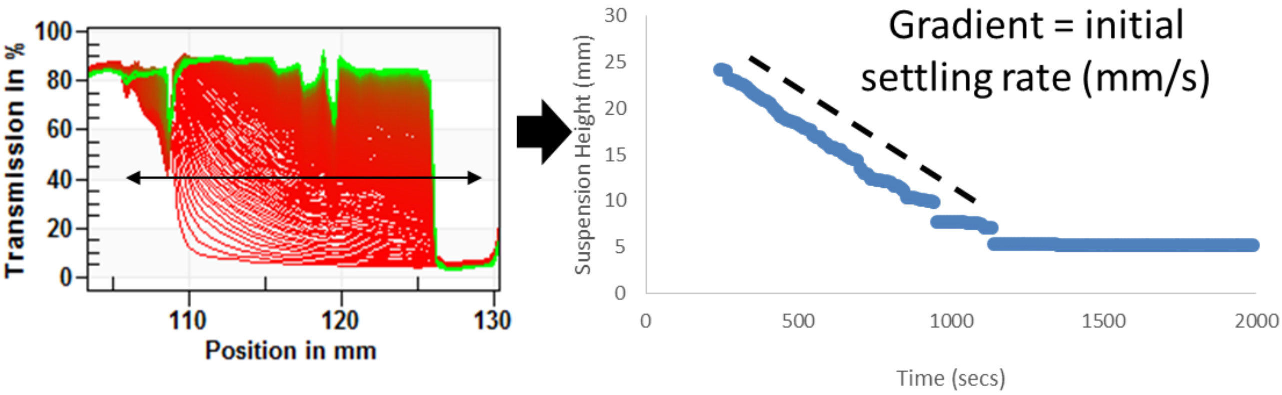

Figure S3. Raw LUMiSizer settling data showing transmission profile taken at 40 % converted to suspension height vs time graph for zirconium molybdate (ZM-a) at 4 vol % in water at 500 rpm.
